# Neural speech tracking in noise reflects the opposing influence of SNR on intelligibility and attentional effort

**DOI:** 10.1162/IMAG.a.126

**Published:** 2025-08-28

**Authors:** Xiaomin He, Vinay S. Raghavan, Nima Mesgarani

**Affiliations:** Department of Electrical Engineering, Columbia University, New York, NY, United States; Mortimer B. Zuckerman Mind Brain Behavior Institute, Columbia University, New York, NY, United States

**Keywords:** selective neural speech tracking, SNR, speech intelligibility, attentional effort

## Abstract

Understanding speech in noise depends on several interacting factors, including the signal-to-noise ratio (SNR), speech intelligibility (SI), and attentional engagement. However, how these factors relate to selective neural speech tracking remains unclear. In this study, we recorded EEG and eye-tracking data while participants performed a selective listening task involving a target talker in the presence of a competing masker talker and background noise across a wide range of SNRs. Our results revealed a non-linear relationship, where neural tracking of the target speech first increased with SNR but then paradoxically decreased as SNR continued to improve. To explain this, we quantified SI behaviorally, estimated attentional effort (AE) using gaze velocity, and measured behavioral performance (BP) via a repeated-word detection task. Our analysis showed that neural tracking of the target speech increased with both SI and attentional engagement. However, when intelligibility reached ceiling levels, selective neural speech tracking decreased as AE declined. Statistical modeling indicated that SI and AE were reliable predictors of neural tracking, while SNR showed no independent contribution after accounting for these factors. Our results demonstrate that improved SNR influences selective neural speech tracking primarily by increasing SI and simultaneously reducing AE, which have opposing effects on neural tracking. These findings underscore the importance of jointly considering these factors in studies of speech perception in noise.

## 1 Introduction

The neural encoding of speech in noise is an essential process that underlies speech comprehension in complex auditory scenes. Various objective and subject-specific perceptual factors influence how the brain processes noisy speech. Objective factors include the signal-to-noise ratio (SNR), representing the physical properties of the acoustic signal and its masking by background noise. Perceptual factors include speech intelligibility (SI) and attentional engagement. SI reflects the listener’s ability to recognize spoken words and depends not only on SNR but also on the listener’s auditory processing capabilities (Nilsson et al., 1994; Sharma et al., 2013). Attentional engagement is, in turn, characterized by two key components: behavioral performance (BP), which reflects how effectively a listener maintains focus, and attentional effort (AE), which involves the cognitive resources expended to focus on the attended talker while ignoring distractions. AE is influenced by the listener’s motivation, fatigue, and overall task difficulty (Bruya & Tang, 2018; Sarter et al., 2006; Strauss & Francis, 2017). While these factors are interconnected, they are mechanistically distinct. SNR is an external, quantifiable measure, whereas intelligibility and attentional engagement are perceptual experiences that vary across individuals, even in identical acoustic settings. Despite extensive research on individual factors such as SNR, intelligibility, and attentional engagement, there remains a critical gap in understanding how these factors jointly relate to selective neural speech tracking.

Past research has extensively studied the neural encoding of speech in noise, emphasizing the role of SNR and SI. Studies demonstrated that increasing SNR generally enhances SI and neural speech encoding (Das et al., 2018; Decruy, Lesenfants, et al., 2020; Ding & Simon, 2013; Lesenfants et al., 2019; Vanthornhout et al., 2018). Others have used varying degrees of visual congruency to modulate SI and examined its impact on neural speech encoding (Crosse et al., 2015; Iotzov & Parra, 2019). Further work has identified different response components that differentially reflect SNR or SI, such as frequency bands (Etard & Reichenbach, 2019; Vanthornhout et al., 2018), temporal components (Decruy, Lesenfants, et al., 2020; Ding & Simon, 2013; Yasmin et al., 2023), and response latency (Yasmin et al., 2023). However, these findings often imply a linear relationship between SNR, SI, and neural speech encoding, overlooking the dynamic interaction among these factors (Krueger et al., 2017). For instance, increasing noise levels under specific conditions can enhance neural speech tracking (Das et al., 2018; Lesenfants et al., 2019), and higher SI does not always correlate with increased neural encoding (Etard & Reichenbach, 2019). Moreover, past research typically used standard speech-in-noise tasks to measure SI, often separating this assessment from the task used to evaluate neural responses. Such standardized SI tasks typically involve asking subjects to repeat short sentences heard in noisy environments (Feng & Chen, 2022; Nilsson et al., 1994; Sharma et al., 2013). This method may only partially capture the complexities of real-world listening due to its limited consideration of attention span and effort, which could vary substantially across both stimuli and task type (e.g., sentence repetition vs. continuous speech comprehension). To better characterize selective neural speech tracking, it is crucial to account for attentional engagement and its interaction with task demands. For example, it has been shown that listeners’ BP in attentive listening tasks also significantly modulates neural speech encoding (Ding & Simon, 2013; Mesgarani & Chang, 2012; O’Sullivan et al., 2015). In addition, SNR can considerably change SI for the target (Brungart et al., 2001) and the AE required to maintain focus on a talker (Cui & Herrmann, 2023; Dimitrijevic et al., 2019; Zekveld et al., 2006). These findings suggest a complex interplay between SNR, SI, attentional engagement, and their impact on neural speech encoding (Devocht et al., 2017; Krueger et al., 2017; Yasmin et al., 2023), highlighting a critical yet unsolved gap in our holistic understanding of how these factors individually and collectively predict neural encoding of speech in noise.

Our study aims to address the need for a comprehensive analysis integrating these factors (SNR, SI, BP, and AE) to fully elucidate their combined impact on neural encoding. We examined neural responses to speech in noise through a multifaceted approach incorporating a finely sampled range of SNRs with small step sizes. We used a repeated-word detection task (Kirchner, 1958; Laffere et al., 2020; Marinato & Baldauf, 2019), designed to continually monitor subjects’ BP during attentive listening tasks . Additionally, we estimated AE by analyzing gaze velocity (Ala et al., 2020; Gopher, 1973) to understand their collective impact on EEG signals. Our findings advance our holistic understanding of noisy speech processing in the brain and have practical implications for designing and parameterizing brain-informed auditory technologies.

## 2 Materials and Methods

### 2.1 Participants

Fourteen native American English speakers (seven males; mean ± standard deviation age, 24.86 ± 4.4 years) with self-reported normal hearing participated in the experiment. The study followed the protocol approved by the Institutional Review Board of Columbia University (Protocol Number: AAAR7230). Participants received monetary compensation and bonuses based on their task performance (repeated-word detection hit rate).

### 2.2 Stimuli and acoustic conditions

In Experiment 1, speech stimuli were drawn from the Connected Speech Test (CST) sentence set (Cox et al., 1987) while in Experiment 2, the stimuli consisted of short, easy-to-understand stories from various sources (e.g., The Moth, Wikipedia). To maintain precise control over acoustic features and ensure consistent coverage across speakers, we synthesized speech using the Google Text-to-Speech API WaveNet (Oord et al., 2016) with four voices: two male voices (‘en-US-Standard-B’ and ‘en-US-Standard-D’) and two female voices (‘en-US-Standard-E’ and ‘en-US-Standard-F’), all synthesized with default speaker parameters.

**Fig. 1.**
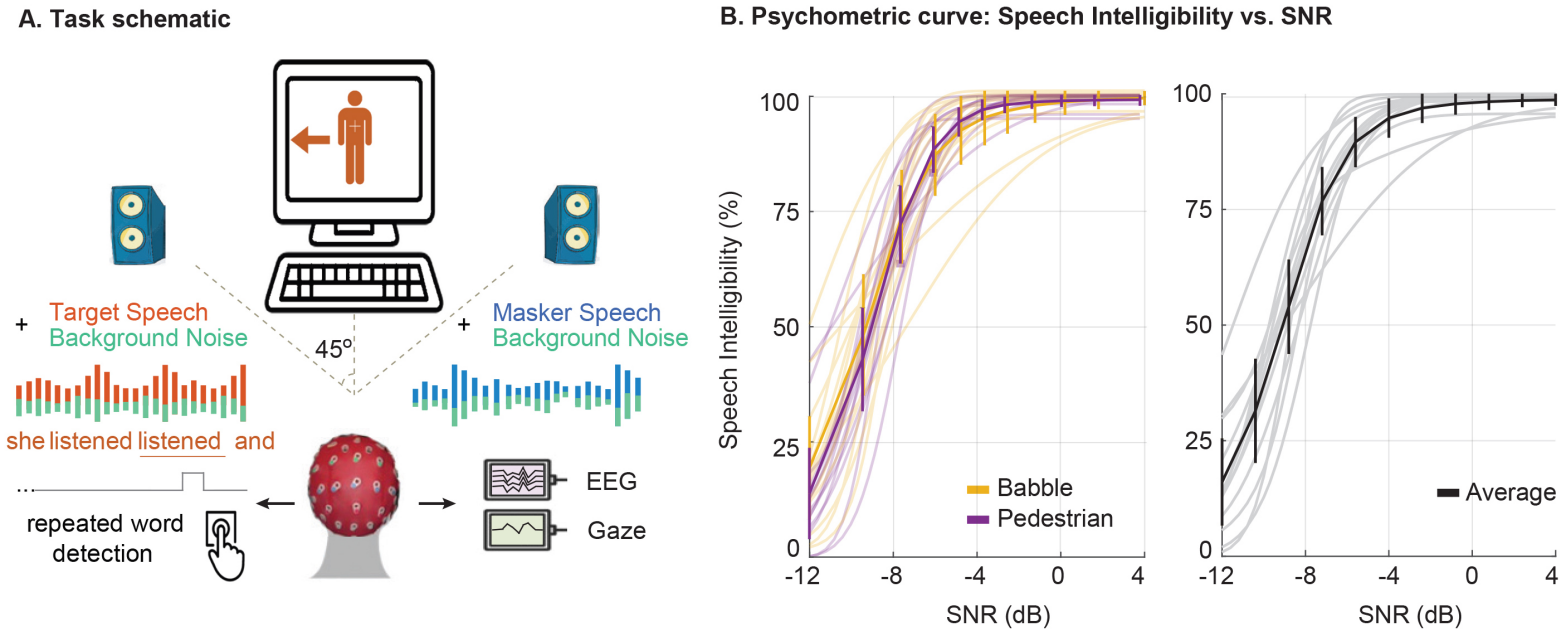
Illustration of the task schematic and psychometric curves. (A) The general task schematic. Subjects were instructed to focus on the target talker while ignoring the masker talker and the background noise. EEG, gaze activities, and button press were recorded while subjects performed the tasks. The subjects press a button when they hear a repeated word in the target stream. (B) Speech intelligibility (SI) is measured using a connected speech task to derive psychometric curves for pedestrian and babble noises. No significant difference appears between the noise types.

During each trial, an arrow was displayed to indicate the target speaker’s direction and guide the listener’s attention to the correct spatial location. The target speaker was masked by a talker of the opposite gender and background noise (Fig. 1A). Speech was presented through two loudspeakers positioned at ±45 degrees relative to the listener. The speakers were spaced approximately 120 cm apart, and the distance from the subject to the monitor was about 60 cm. Target stimuli were normalized to 65 dB SPL and mixed with the competing masker speech and background noise at various SNR, ranging from -12 dB to +4 dB.

To ensure the generalizability of our findings across different real-world acoustic conditions, we included two types of background noise: 10-talker babble noise from the AzBio test (Spahr et al., 2012) and pedestrian street noise from the CHiME3 corpus (Barker et al., 2015). To ensure that masking was consistent and not driven by transient, attention-capturing events, we manually removed salient sounds such as car horns, high-pitched braking, and intelligible background speech. The resulting noise was presented equally to both loudspeakers. Babble noise consists of overlapping speech, making it more similar to the target, whereas pedestrian noise contains non-speech environmental sounds that are more distinct from the speech signal. Including both noise types helped confirm that our results were not dependent on a specific masking condition.

We used four synthetic voices (two male and two female), and each trial featured a target–masker pair composed of speakers of opposite gender. These voice pairings were fully crossed with two background noise types (babble and pedestrian), yielding 16 unique target–masker–noise combinations (4 target voices × 2 opposite-gender maskers × 2 noise types). Trials were evenly distributed across these combinations to avoid bias from specific speakers or noise conditions.

### 2.3 Experiment procedures

#### 2.3.1 Experiment 1: measuring intelligibility by connected speech test

Speech intelligibility (SI) was assessed using the CST (Cox et al., 1987). Participants listened to 16 sets of short, connected sentences (10 sentences per set) on familiar everyday topics. Participants were instructed to repeat aloud the words they heard. Their responses were recorded by the experimenters, and intelligibility scores were computed for each SNR level. These scores were used to derive individual psychometric SI curves (details in Section 2.5.2).

To help the identification of target speech in trials with very low SNRs, each trial began with a leading sentence, “repeat after me,” during which the masker speech and background noise gradually increased from silence to their designated levels.

#### 2.3.2 Experiment 2: multi-talker speech-in-noise perception test

American English podcast stories were presented with the same setting as experiment 1 (Fig. 1A). Participants listened to 160 trials of stories (average length ~35 s) with the target speaker cued on screen, and they needed to press the button whenever they heard a repeated word from the target speaker. After each round of 16 trials, experimenters calculated the button responses to the repeated words (repeated-word hit rate) and reported them to subjects as feedback. To ensure the subjects attended to the content, experimenters also asked them to summarize the stories they heard.

During experiment 2, subjects were instructed to fixate on a visual cross and minimize head movement during each trial to facilitate EEG recording and eye tracking. To help the identification of target speech in trials with very low SNRs, each trial began with a 3-s window during which the masker speech and background noise gradually increased from silence to their designated levels. During this period, only the clean target speech was initially audible. This time window was excluded from all analysis.

### 2.4 Data acquisition and preprocessing

During the experiment, 64-channel EEG, button responses to the repeated words, and eye-tracking data were recorded for each trial. The button responses and EEG were recorded by g.HIAMP system (g.tec, Australia). Eye tracking data were calibrated and acquired from Tobii Pro Nano (Tobii, Sweden). All data were streamed from Simulink (Mathworks, MA, USA) at 1200 Hz with a 60 Hz notch filter. Afterward, EEG data were downsampled to 100 Hz with an anti-aliasing filter. Channels with unusual standard deviations were automatically detected and replaced using spherical interpolation of the remaining channels (Delorme & Makeig, 2004; Kang et al., 2015; Perrin et al., 1989).

Speech envelopes for both target and masker speakers were firstly extracted by a nonlinear, iterative (NLI) method (Horwitz-Martin et al., 2016) and secondly downsampled to 100 Hz to match with the EEG recordings. Finally, each envelope was z-scored to zero mean and unit variance. Blink detection and gaze tracking were completed and preprocessed automatically by Tobii Pro SDK (Tobii, Sweden) with a sampling frequency of 60 Hz. The gaze coordinates were normalized to (0,0) and (1,1) within the screen.

### 2.5 Measurement of objective and perceptual factors

#### 2.5.1 Speech objective factor: signal-to-noise ratio (SNR) of target speech

In both experiments, the target speech volume was presented at a fixed volume of 65 dB SPL while the levels of masker speech and background noise were jointly adjusted to create SNRs ranging from -12 dB to +4 dB. This range was empirically chosen to span the full intelligibility spectrum (0%–100%) for most normal-hearing listeners. A total of 80 distinct SNR values were used, each applied under both noise conditions (babble and pedestrian).

The SNR of the target speech was defined relative to all competing auditory inputs, including both the masker speech and the stereo background noise. To ensure consistency, the masker and each channel of the noise were presented at equal sound pressure levels. This relationship is expressed in the following formula:

$$\begin{array}{l} SNR_{\mathrm{target}}=L_{\mathrm{target}}-L_{\mathrm{total}\;\mathrm{noise}}\\ \;\;\;\;\;\;\;\;\;\;\;\;\;\;\;\;\;\;=L_{\mathrm{target}}-10\log_{10}(P_{\mathrm{masker}}+2P_{\mathrm{noise}})\\ \;\;\;\;\;\;\;\;\;\;\;\;\;\;\;\;\;=L_{\mathrm{target}}-10\log_{10}3P_{\mathrm{masker}}\\ \;\;\;\;\;\;\;\;\;\;\;\;\;\;\;\;\approx L_{\mathrm{target}}-L_{\mathrm{masker}}-4.77 \end{array}$$ (1)

where ***L*** is the sound pressure level (SP*L*); ***P*** is the power of stimuli; $L_{target}$ is 65 dB; and $P_{masker\;}$ and $P_{noise\;}\;$ stay identical during the setting, so do $L_{masker}$ and $L_{noise}$ .

To disentangle the correlated effects of SNR and SI, we included trials in which SI was at or near ceiling, typically reached at SNRs between -6 dB and -2 dB, depending on the individual. In these conditions, speech was fully intelligible, but SNR continued to vary, allowing us to assess the independent contribution of SNR to selective neural speech tracking beyond its effect on intelligibility.

#### 2.5.2 Speech perceptual factor: speech intelligibility (SI)

SI was measured using the CST (Cox et al., 1987). In Experiment 1, experimenters manually filed the subjects’ word recall accuracy for each SNR bin. Then, the psychometric curve between SNR values and word recall accuracy was fitted using *psignifit* toolbox, which implements either the maximum-likelihood method described by (Wichmann & Hill, 2001a, 2001b). In this implementation, we used logistic function as the kernel of the sigmoid, originally proposed by (Festen & Plomp, 1990), and later generalized to allow slight variations in output range for improved robustness (Wichmann & Hill, 2001a):

$$SI\;=\;lw\;+\;\frac{up-lw}{1+exp^{-gr*\left(SNR-ths\right)}}$$ (2)

where ***lw*** is the lower bound (approaching 0); ***up*** is the upper bound (approaching 1); ***gr*** is the growth rate; ***SNR*** is the signal-to-noise ratio of the target speech, range from -12 to +4 dB; and ***ths*** is the speech reception threshold when SI = 50%, defined in the range of SNR.

We fitted individual psychometric curves for each subject and noise type using equation (2). As no significant differences were found across noise types, we averaged the two curves for each participant. The resulting subject-specific SI curves were used to derive SI values for each trial in Experiment 2 (see Fig. 1B). The black curve in Figure 1B shows the group average, while gray curves show individual subject fits, with error bars indicating standard deviation across participants at each SNR level.

For each psychometric curve, we defined the regressed upper bound as the ceiling SI, representing the maximum SI a subject could achieve. In Experiment 2, we purposely selected SNRs above the minimum SNR required for ceiling SI, as described in Section 2.5.1.

#### 2.5.3 Attentional engagement metrics

##### 2.5.3.1 Behavioral performance (BP): single-trial repeated-word hit rate (HR)

We used the single-trial repeated-word hit rate (HR) to measure subjects’ BP during each trial (Kirchner, 1958; Laffere et al., 2020; Marinato & Baldauf, 2019) as an index of attentional engagement, without imposing additional physiological load on working memory. For simplicity, we selected semantically important keywords as repeated words, excluding articles, prepositions, and conjunctions. Three repeated words were inserted into each trial, resulting in an HR range of [0, ⅓, ⅔, 1]. A higher HR corresponds to better behavioral performance. However, as a behavioral outcome, HR is a composite measure, necessarily influenced by the perceptual challenges of the task, such as SNR and SI. Therefore, when using HR to infer a listener’s attentional state, comparisons should only be made between trials with similar SNR and SI.

To help ensure that participants were not focused solely on detecting repeated words, we included brief comprehension questions at the end of each block. These questions prompted participants to recall the general content of the stories, encouraging broader engagement with the speech beyond acoustic-level cues. Although responses were not tied to compensation, they served as a qualitative check on comprehension and sustained attention.

##### 2.5.3.2 Attentional effort (AE): gaze velocity (GV)

Attentional engagement is associated with suppressed irrelevant physiological activities, evidenced by reduced oculomotor activity, indexed by reduced saccade or micro-saccade rate, reduced blink rate, as well as prolonged fixation (Abeles et al., 2020; Braga et al., 2016; Contadini-Wright et al., 2023; Cui & Herrmann, 2023). Oculomotor activity, with its anatomical overlap with the attention-related network (Corbetta et al., 1998), is proposed as a metric for evaluating AE (Ala et al., 2020; Gopher, 1973). In this study, we use averaged GV to quantify AE for each trial, as it reflects the overall intensity of oculomotor activity, including saccades and micro-saccades. A higher GV, indicating more frequent oculomotor activity, suggests reduced AE (Ala et al., 2020; Gopher, 1973)

The gaze coordinates were recorded and normalized between (0,0) and (1,1) by the Tobii Pro Nano screen-based eye tracker. To calculate actual gaze angular velocity, we first restored relative coordinates to screen size, then computed and averaged the absolute value of the derivative of gaze coordinates over time within each trial. Finally, using the (approximately) 0.6 m distance from the subject’s seat to the screen, we calculated GV using trigonometric functions (Diaz et al., 2013).

### 2.6 Measuring selective neural speech tracking

We applied canonical correlation analysis (CCA) algorithms for target speech decoding (Dähne et al., 2015; de Cheveigné et al., 2018). Speech envelopes were extracted from both the target and masker speech. Speech envelopes and EEG signals were first downsampled to 100 Hz. Next, the stimuli and neural recordings were windowed into overlapping receptive fields. Time-lagged matrices were then generated for envelopes and EEG signals, with 400 ms receptive fields for EEG and 200 ms for speech envelopes. Finally, CCA-based linear models were trained separately for the target and masker speech using a leave-one-trial-out cross-validation approach for each subject.

The mapping between stimuli and neural responses was quantified by the trained model and evaluated using Pearson’s correlation between transformed stimuli and neural responses. Since the target and masker stimuli are encoded differently in the brain (Ding & Simon, 2012a; Mesgarani & Chang, 2012), we computed this correlation for both the target (rT) and masker (rM) speech. We defined the difference between these correlations as rD (rD = rT - rM) which quantifies the relative strength of selective neural tracking of the target speech due to selective attention.

To investigate neural modulation patterns under different speech conditions, we estimated temporal response functions (TRFs) of the target speech using regularized linear regression (Ding & Simon, 2012b; Lalor et al., 2009). This approach minimizes the mean-square error between the actual and predicted neural responses. The training and prediction processes were performed for each subject using a leave-one-trial-out cross-validation approach, implemented with the mTRF toolbox (Crosse et al., 2016).

### 2.7 Statistical analysis

Unless otherwise noted, error bars in all figures represent the standard error of the mean (SEM).

**Fig. 2.**
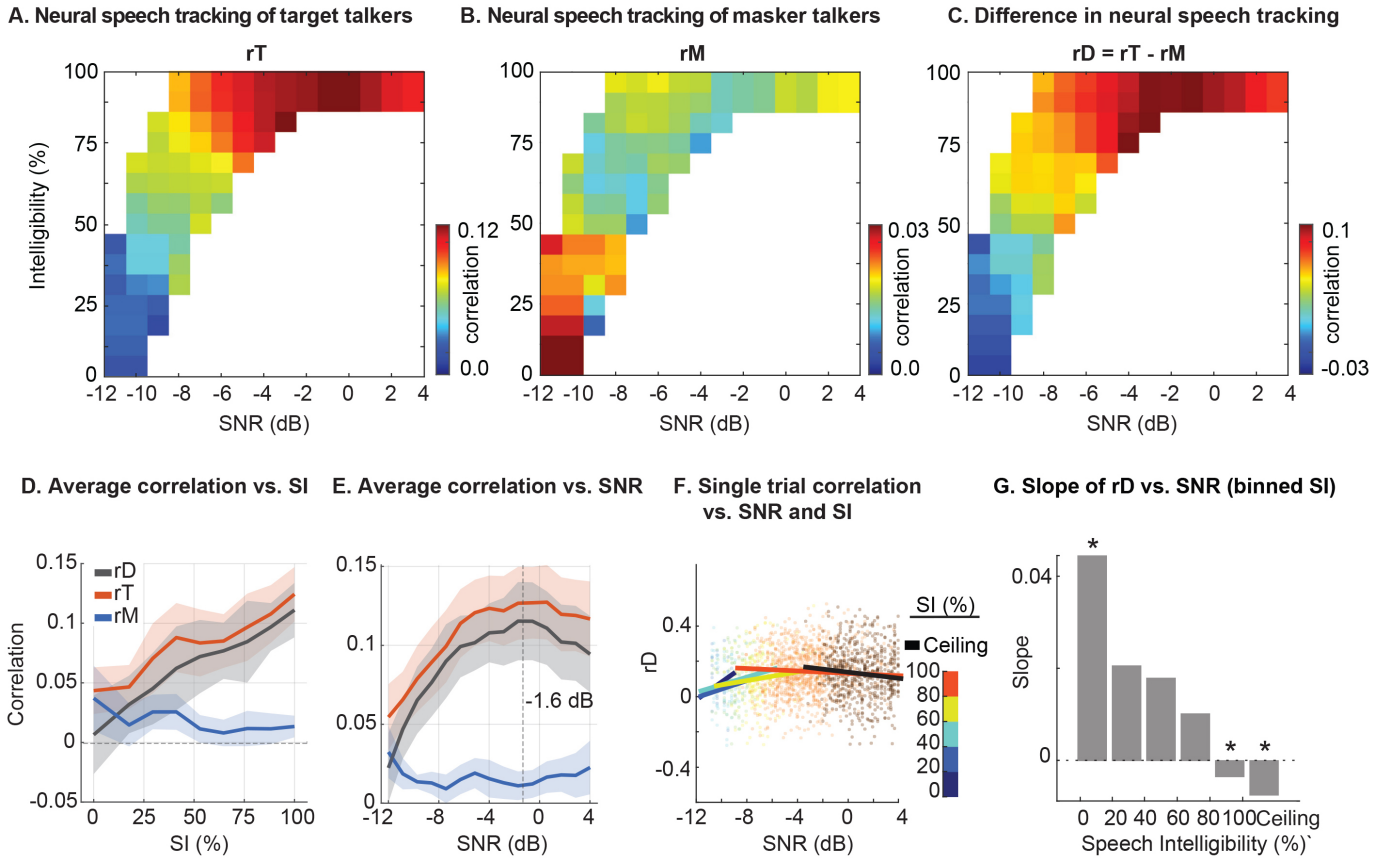
Neural speech tracking as a function of SNR and SI. (A) Correlation of the target neural speech tracking (rT) across different SNR and SI values; each bin is color-coded by the averaged rT. (B) Correlation of masker neural speech tracking (rM) across different SNR and SI values. Each bin is color-coded by the averaged rM. (C) Difference in neural speech tracking (rD = rT-rM) between target and masker streams. Each bin is color-coded by the averaged rD. (D) Subject-averaged neural speech tracking correlations (rT, rM, and rD) plotted against SI. Error bars represent the SEM across subjects. (E) Subject-averaged neural speech tracking correlations (rT, rM, and rD) plotted against SNR. Error bars represent the SEM across subjects. (F) rD plotted across SNR values for different SI bins. Linear fits are computed within each SI bin and averaged across subjects. Data points and regression lines are color-coded by SI levels. (G) Slopes of the rD-SNR relationship for each SI bin, derived from LMEs with subject-level random effects controlled. Bars represent the average combined effect (fixed + random effects) across subjects. Asterisks denote slopes with significant fixed effects (t-test, p < 0.05).

**Fig. 3.**
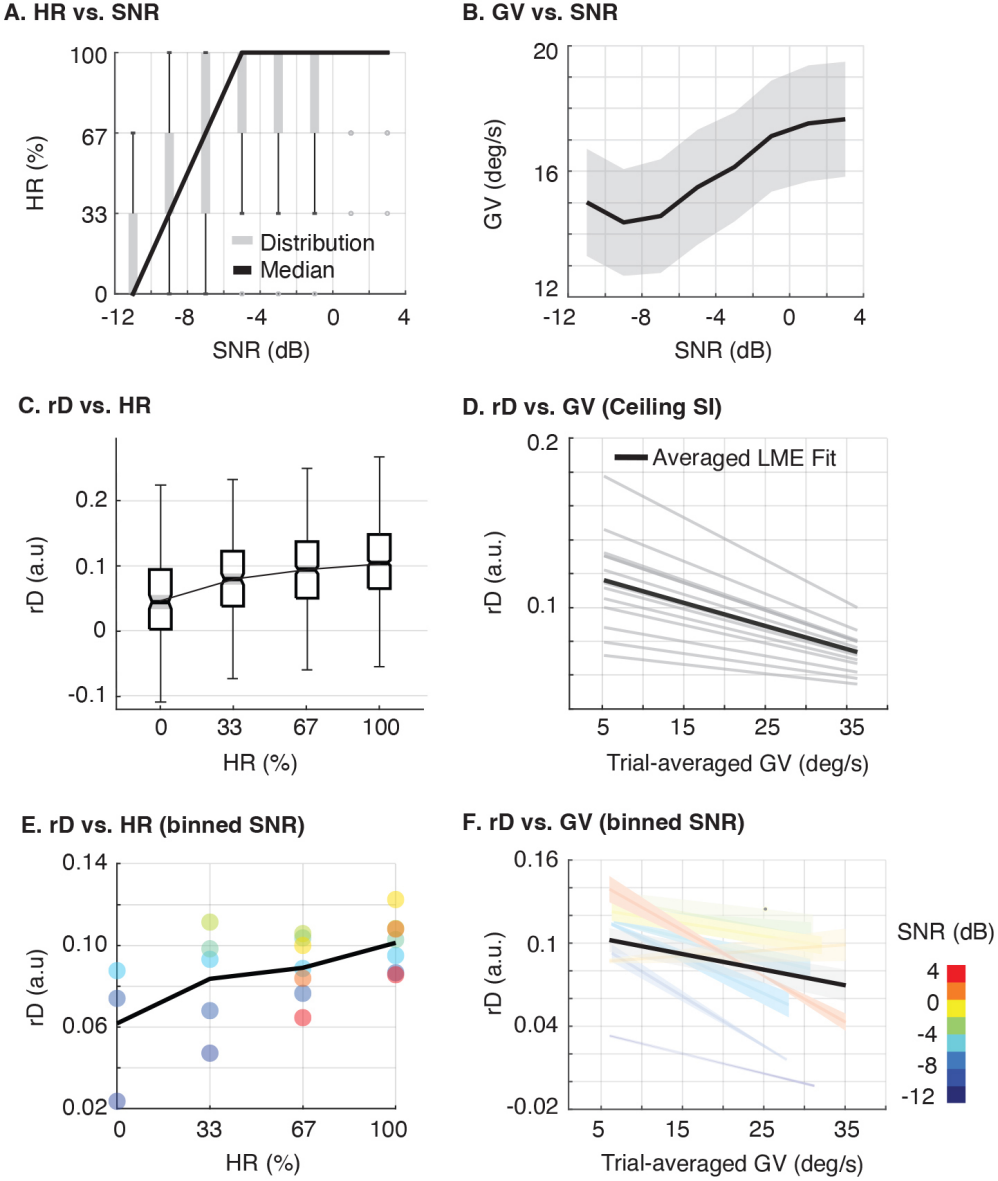
Behavioral performance (BP) and attentional effort (AE). (A) Single-trial repeated-word hit rate (HR) increases with SNR. (B) Gaze Velocity (GV) also increases with SNR, indicating reduced AE at higher SNRs. (C) The relationship between the selective neural tracking of target speech (rD) and HR. Trials are grouped by HR levels, with medians and confidence intervals shown. Significant differences between groups were identified using a Kruskal-Wallis test with Bonferroni-corrected Dunn’s post hoc comparisons (p < 0.05). (D) In high-intelligibility (ceiling SI) trials, GV is negatively associated with rD, as shown by LME modeling with subject-level control. The black line shows the average effect across subjects; gray lines indicate individual fits. (E) The positive relationship between HR and rD persists when modeled separately for each SNR bin. (F) Similarly, the negative relationship between GV and rD persists across all SNR bins. The black line shows the average effect from the LME model, confirming that GV consistently predicts lower rD regardless of task difficulty.

**Fig. 4.**
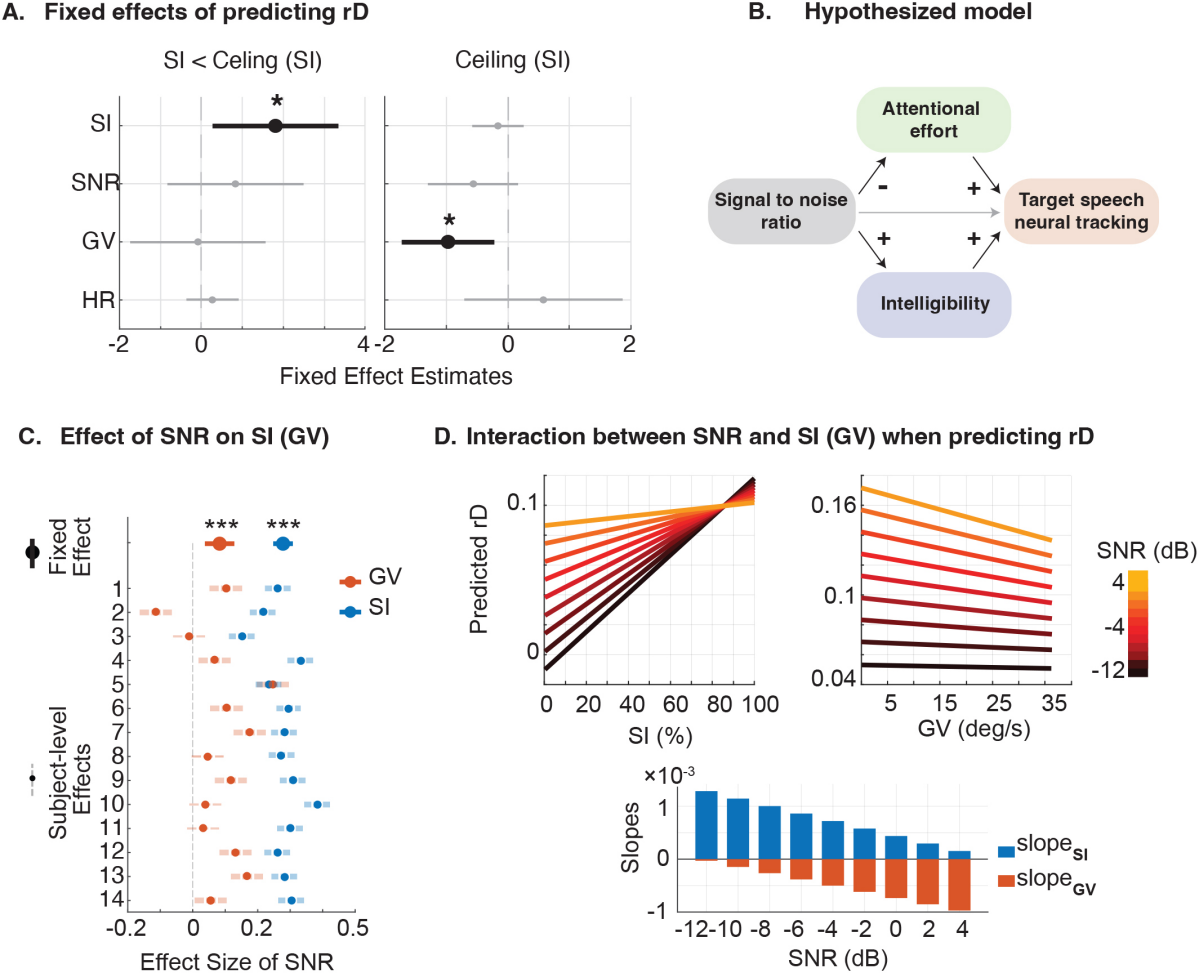
Predictive contributions and interactions among all factors. (A) Fixed effects from LME predicting rD under two conditions: SI < ceiling and SI at the ceiling. Subject-level effects are controlled. Predictors with significant fixed effects are highlighted in bold (*p < 0.05; ***p < 0.001). (B) Conceptual model illustrating hypothesized interactions between SNR, SI, GV, and rD. (C) Modulation of SI and GV by SNR, modeled using LME with subject-level control. Both fixed and random effects are shown. Predictors with significant fixed effects (t-test, p < 0.05), and subjects with significant random effects (t-test, p < 0.05), are highlighted in bold. (D) Interaction between SNR and SI or GV in predicting rD, using subject-averaged linear models. The bar plot below shows the average slopes between rD and SI (or GV) at each SNR level.

To examine the effects of experimental factors on rD, we used linear mixed-effects models (LME, Pinheiro & Bates, 2009; Snijders & Bosker, 1999) incorporating both fixed effects (SNR, SI, GV, etc.), which reflect population-level effects of these variables, and random effects at the subject level, which account for variability across participants. We fitted curves reflecting the combined fixed and random effects of these models, and statistical significance was assessed using t-tests on fixed and random effects (p < 0.05). The resulting significance was directly reported in Figure 4 and used to determine the significance of slope trends in Figures 2 and 3.

**Fig. 5.**
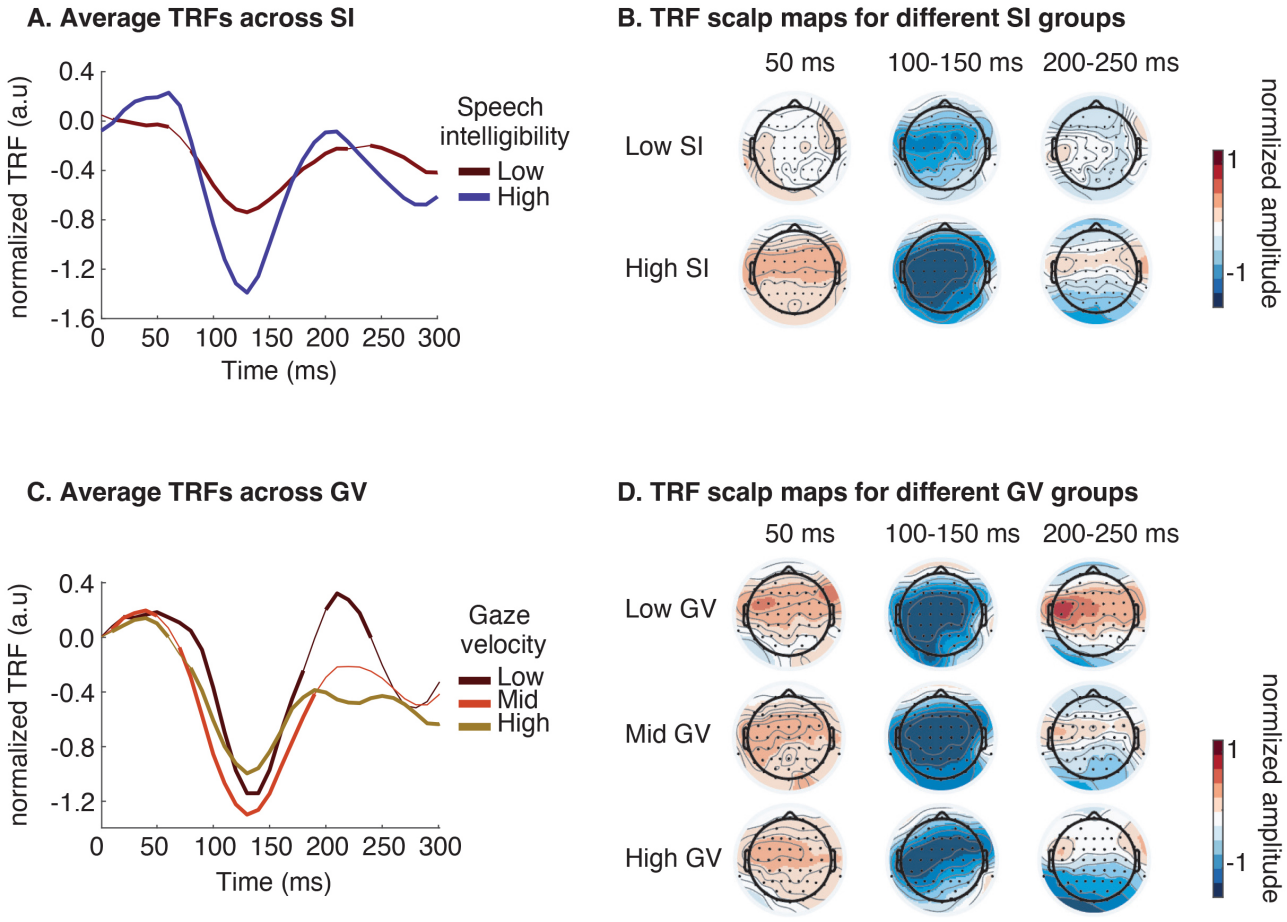
Normalized temporal response functions (TRFs) under different SI or GV levels. (A) The normalized TRFs under low (<50%) and high (>50%) SI. TRFs are averaged across all EEG channels. Significant temporal components (compared to the chance level, Bonferroni-corrected t-test, p<0.05) are marked with thick lines. (B) The topography of normalized TRFs amplitude at three critical time points (50 ms, 100~150 ms, and 200~250 ms), under different levels of SI. (C) The normalized TRFs under low (<33%), mid (33%-67%), and high (>67%) GV. TRFs are averaged across all EEG channels. Significant temporal components (compared to the chance level, Bonferroni-corrected t-test, p < 0.05) are marked in thick lines. (D) The topography of normalized TRFs amplitude at three critical time points (50 ms, 100~150 ms, and 200~250 ms), under different levels of GV.

To identify significant TRF components (highlighted with thick strokes in Figure 5), we performed a permutation test. Specifically, we permuted the speech envelope and neural responses 1000 times to generate a null distribution representing chance-level alignment. The observed TRFs were then compared to this null distribution at each time point using a t-test. Time points with Bonferroni-corrected p-values less than 0.05 were considered statistically significant.

## 3 Results

Fourteen participants performed a selective listening task (Fig. 1A), attending to a target speaker while ignoring a masker speaker and background noises across various SNR levels, ranging from -12 dB to 4 dB. Throughout the experiment, we recorded 64-channel EEG to measure neural responses, along with gaze velocity (GV) and repeated-word hit rate (HR) to capture behavioral responses. From these recordings, we defined our key perceptual factors. Speech intelligibility (SI) was assessed for each participant before the main task using the Connected Speech Test (CST) (Cox et al., 1987) (Fig. 1B). For each trial, behavioral performance (BP) was measured by the hit rate (HR) for the target speech, and attentional effort (AE) was inferred from GV. The aim of this study was to examine the interaction between these factors and their collective impact on selective neural speech tracking in noisy conditions.

### 3.1 Distinct associations of SNR and SI with selective neural speech tracking

To measure the strength of neural tracking for target and masker speech, we trained CCA-based linear models to quantify neural speech tracking for each talker. We computed Pearson’s correlation between the transformed speech envelope and neural recordings to measure the strength of neural speech tracking for the target (rT) and masker (rM) speech. The difference in correlation between target and masker speech (rD *=* rT *–* rM) was used as a single measure to reflect how well participants selectively followed the target speech while suppressing the masker speech.

To accurately assess the impact of SNR and SI on target and masker neural speech tracking, it is crucial to distinguish between these two highly correlated factors. We addressed this by analyzing their effect on neural speech tracking as a function of both SI and SNR. Our analysis revealed distinct patterns in how SNR and SI are associated with neural speech tracking. Figure 2A-C shows the averaged neural tracking correlations across subjects for target (rT) and masker speech (rM), as well as their difference (rD), across different SI and SNR values. We found that while increasing SNR and SI are generally associated with increased rT (enhanced neural tracking of the target speech) and decreased rM (suppressed neural tracking of the masker speech), this pattern changed when SI is sufficiently high. Specifically, under high SI conditions (i.e., SI exceeding around 80%, as shown in Fig. 2A), increasing SNR is associated with reduced rT and increased rM, indicating decreased neural tracking of the target speech while increasing the tracking of the masker speaker.

The average plots across SI and SNR in Figure 2D and 2E further illustrate these effects, showing that SI has a nearly monotonic relationship with neural speech tracking, while SNR’s impact on these values is observed to reverse beyond approximately -1.6 dB. This indicates that in easier listening conditions, when the target talker is highly intelligible, increasing the SNRs can paradoxically reduce the selective neural speech tracking as reflected in reduced rT and increased rM. In Figure 2F, we quantized SI into 6 bins from 0 to ceiling SI, each denoted by a different color. Trials categorized as ‘Ceiling SI’ are marked in black and represent instances where SI approached its regressed upper bound on individual psychometric curves. Figure 2G shows the linear relationship between SNR and rD within each bin of SI, using LMEs that include both fixed and random subject-level effects. Significant slopes were marked with asterisks based on the fixed effect (t-test, p < 0.05). As SI increases, the slope between SNR and rD shifts from positive to neutral, and eventually to negative.

In summary, these results demonstrate that while SNR and SI are strongly correlated, they have distinct and at times opposing associations with the selective neural speech tracking. The observed reduction in rD at high SNRs may seem counterintuitive, as higher SNRs typically improve SI. However, in this upper SNR range (typically above -1.6 dB), SI approaches the ceiling of the psychometric function for normal hearing listeners, such that further increases in SNR yield little to no gain in intelligibility. At the same time, reduced task difficulty in these favorable conditions could potentially modulate attentional engagement, for example, by lowering the effort required to maintain focus, which, in turn, contributes to reduced neural tracking. We examine this interaction in more detail in the next section.

### 3.2 Increased SNR reduces attentional effort and selective neural speech tracking

To investigate why rD decreases at high SNRs, we examined how SNR affects the two components of attentional engagement: BP and AE.

First, we analyzed BP, measured via HR As shown in Figure 3A, HR improves with SNR but reaches a ceiling around -5 dB. This suggests the BP metric cannot, on its own, explain the changes in rD in the upper SNR range.

In contrast, AE, measured via GV showed a different pattern. (Note that lower GV indicates higher AE). Figure 3B shows that GV significantly *increases* at higher SNRs, particularly where speech is highly intelligible. This indicates that the AE required to maintain focus substantially decreases when listening conditions are easy.

Having established that both attentional engagement metrics are modulated by SNR, we next investigated their direct impact on rD (Fig. 3C, 3D). Figure 3C shows that the HR significantly modulates rD. A Kruskal-Wallis test confirmed significant differences between the HR groups (p < 0.05), with post-hoc comparisons performed using a Bonferroni-corrected Dunn’s test. Higher HR was found to be associated with higher rD. However, this change is not continuous due to the discrete nature of the behavioral response (three repeated words in each trial). In contrast, AE, measured by GV, exhibits a more stable effect on rD. Figure 3D shows that in highly intelligible trials with ceiling SI, reduced AE, as indicated by increased GV, predicts lower rD. This negative relationship between rD and GV was confirmed using LME in Figure 3D, showing significant negative combined effects (fixed + random effects) across subjects, with subject-level variance properly accounted for. This finding further supports that the increase of GV in intelligible trials shown in Figure 3B is related to a reduced rD.

To determine if the influence of attentional engagement on rD is consistent across listening conditions, we repeated the LME analysis within discrete SNR bins (Fig. 3E, 3F). The results confirm that the distinct relationship for each metric persists across all SNR levels. Specifically, we observed a consistent positive relationship between HR and rD (Fig. 3E), and a consistent negative relationship between GV and rD (Fig. 3F). Therefore, while the effect of AE on rD is most pronounced at high SNRs, both metrics of attentional engagement show persistent, opposing correlations with rD regardless of the overall task difficulty.

In summary, Figure 3 demonstrates that AE decreases as SNR increases, meaning subjects exert less effort in easier trials, which also corresponds to decreased selective neural tracking of the target speech.

### 3.3 Modeling the interactions between SNR, speech intelligibility, and attentional effort on selective neural speech tracking

Given that our previous results indicate that multiple interacting variables influence rD, we fitted a linear mixed-effects model to predict rD from these variables, controlling for subject-level effects. Separate models were fitted under two conditions: (a) trials where SI was below ceiling (Adjusted R² = 0.1384, likelihood ratio test vs. null model: p < 1e-5), and (b) trials at ceiling SI (Adjusted R² = 0.0143, likelihood ratio test vs. null model: p = 0.016). The fixed effects from these models are shown in Figure 4A, with statistically significant predictors (p < 0.05, t-test on fixed effects) highlighted in bold. This analysis shows that SI is a positive predictor of rD when not at ceiling, while AE emerges as a significant negative predictor when SI is at its maximum. Interestingly, after accounting for the effects of SI and AE, the models show no significant unique contribution from SNR or HR to the prediction of rD. Given the strong correlation between SNR and SI in this paradigm, this finding suggests that the influence of SNR on selectively tracking of target speech is largely mediated by its impact on speech intelligibility and attentional effort. Specifically, our results support a model where increasing SNR acts through two pathways with opposing effects: it improves SI, which enhances neural tracking, while simultaneously reducing the required AE, which diminishes neural tracking. This opposing effects of SI and AE on rD could explain the non-linear relationship observed in Figure 2E. This interaction is shown in Figure 4B.

Figure 4C and 4D further support the proposed model. Figure 4C demonstrates the modulation of SI and GV by SNR using LME modeling (with subject-level effects controlled; significance determined by t-test, p < 0.05). SNR showed a significant positive effect on SI at the group level, and this trend was consistent across most participants. In contrast, SNR had a significant negative fixed effect on GV, with some variability across subjects.

Figure 4D validates the two modulation pathways proposed in Figure 4B (LME predictions for each SNR level, controlling for subject level and all irrelevant factors). SI positively predicts rD, but its impact weakens as SNR increases, likely due to SI approaching the ceiling at higher SNRs. In contrast, GV negatively predicts rD across SNRs, with its negative impact becoming more pronounced at high SNRs. This steeper slope suggests that AE plays an increasingly significant role in shaping the neural target tracking at higher SNR, particularly after SI is at ceiling level. This interplay between two opposing factors, the weakening positive effect of SI and the strengthening negative effect of AE, jointly shapes the overall non-linear relationship between SNR and rD. This dynamic is summarized in the bar plot at the bottom of Figure 4D, which displays the LME-derived slopes for each factor’s relationship with rD at various SNR levels.

### 3.4 Temporal and spatial dynamics of neural responses under varying speech intelligibility and attentional effort

To investigate how the timing and spatial distribution of neural response patterns change under different SI and GV conditions, we calculated the temporal response functions (TRFs, Ding & Simon, 2012b; Lalor et al., 2009) for target speech in different listening conditions. TRFs capture the brain’s temporal dynamics in response to continuous auditory stimuli, reflecting the relationship between the EEG signal and the speech envelope over lags at different electrodes. Normalized TRFs for target speech are shown in Figure 5, with Figure 5A/C showing channel-averaged TRFs and Figure 5B/D showing the scalp map of TRFs. Significant TRF components are indicated by bold lines. These were identified by comparing observed TRFs to a null distribution generated by permuting the alignment between speech envelopes and neural responses 1000 times. The time points with Bonferroni-corrected p-values below 0.05 were considered statistically significant.

In Figure 5A-B, the group with low SI (< 50%) exhibits weaker early components TRF_50_ (positive, around 50 ms) across the scalp, especially in the temporal and central regions. Additionally, the low SI group shows reduced TRF_100_ (negative, around 100 ms) and TRF_200_ (positive, around 200 ms) components, which are related to attentional engagement, indicating reduced selectivity for target speech and less suppression of masker speech components (Ding & Simon, 2012b; Fiedler et al., 2019).

AE also impacts the TRFs, as shown in Figure 5C-D. While the acoustics-modulated early components TRF_50_ remain consistent across different GV, a significant difference emerges for higher-level, attention-related components around TRF_100_ and TRF_200_ (Fig. 5C, 5D). In trials with lower AE (high GV, GV>66.7% percentile), TRF_100_ responses decrease in posterior electrodes (Fig. 5D). For TRF_200_, only the group with low GV (GV<33.3% percentile) shows strong activation in the anterior and central areas.

## 4 Discussion

We demonstrate that the selective neural tracking of target speech is associated with both objective (SNR) and subject-specific perceptual factors (SI, AE, and BP) in distinct ways. As SI increases, the positive effect of improving SNR on rD diminishes. Specifically, in favorable listening conditions where speech is sufficiently intelligible, further increases in SNR reduce the selective neural speech tracking. We propose that this decrease is driven by the reduced AE required to focus on the target speech. Our findings show that GV, a measure proposed for quantifying AE, effectively explains this reduction in selective neural speech tracking, particularly when compared to BP. Together, our findings suggest a complex interaction between SI and AE, mediated by SNR, in shaping the neural representation of speech in noise.

### 4.1 Distinct neural correlates of SI and SNR in selective neural speech tracking

Although SNR and SI are inherently correlated, especially at the individual level, our experimental design and modeling approach help address this challenge. Subject-specific variability in psychometric functions allows the same SNR value to correspond to different SI levels across participants. Furthermore, by sampling a wide SNR range, including regions where SI saturates, we were able to observe the dissociable effects of SNR and SI on rD.

Through this approach, our findings reveal distinct interactions of SNR and SI with the selective neural tracking of target speech, as measured by EEG (Fig. 2). Importantly, increasing SNR under high SI conditions can reduce the selective neural tracking of target speech. This effect has been previously implied but not further explored by (Das et al., 2018; Lesenfants et al., 2019), who observed decreased selective neural speech tracking from mildly noisy to clean conditions. Previous studies, including those by (Das et al., 2018; Decruy, Lesenfants, et al., 2020; Vanthornhout et al., 2018), primarily investigated the impact of SI on selective neural tracking at different SNRs but overlooked potential differences in neural modulation mechanisms between SI and SNR. Our results highlight the importance of differentiating the effects of SI and SNR.

Objective acoustic factors, like SNR, drive bottom-up processing in speech perception. In contrast, SI is shaped by both listeners’ bottom-up perception and top-down processing capabilities and strategies for allocating cognitive resources or restoring factors masked by noise (Raghavan et al., 2023). This distinction on processing direction highlights how SNR and SI contribute differently to neural processing. For example, the activity in brain regions responsible for top-down processing is increased when bottom-up processing was impaired by degraded speech (lower SNRs) (Zekveld et al., 2006). Several previous studies also suggested different modulations of selective neural speech tracking by objective and perceptual speech factors. Previous research has shown that SI and SNR have different impact patterns on TRFs: the variation of SI induces changes on latency of the TRFs, and SNR non-linearly modulates the amplitude of the TRFs (Yasmin et al., 2023). In contrast, the relationship between selective neural speech tracking and SI does not exhibit such non-linearity (Decruy, Lesenfants, et al., 2020), Moreover, neural speech tracking of target and non-target speech, as well as their difference, show slightly dissimilar correlations with SNR and SI, but this mismatch has remained unexplained (Nogueira & Dolhopiatenko, 2022). Anatomically, attentively listening to less intelligible stimuli without modulating SNR engages the dorsal auditory pathway and slightly lower activation on STG, compared to clear stimuli, which also suggests the distinct neural correlates with SNR and SI (Hervais-Adelman et al., 2012). Our findings go beyond these observations by dissociating the interplay between SI and SNR. We provide evidence that the contribution of SNR to selective neural speech tracking is largely mediated by its influence on AE and SI. This supports the notion that SNR’s contribution is primarily indirect, as also suggested by (Etard & Reichenbach, 2019).

It is important to note that our measure of neural speech tracking is based on EEG recordings, which reflect scalp potentials from large populations of neurons but do not provide the fine-grained detail available from invasive or single-neuron recordings. Studies using invasive techniques in animals and humans have shown that noise-invariant representations gradually develop along the auditory pathway (Kell & McDermott, 2019; Mesgarani et al., 2014; Rabinowitz et al., 2013), with lower areas representing the noise and higher areas filtering it out. Our findings highlight how the combined effects of these interactions manifest in scalp EEG signals, which is critical as EEG is the most widely used measure to study speech in noise in normal hearing, hearing-impaired, and aging individuals (Di Liberto et al., 2022; Fuglsang et al., 2020; Mesik et al., 2021).

### 4.2 The role of attentional engagement in selective neural speech tracking

Attentional engagement plays a crucial role in how the brain selectively tracks the target speech in noise and suppresses the masker. For instance, the acoustic characteristics of speech, most notably the SNR, directly influence the level of attentional engagement required from the listener. In our study, we followed a framework where attentional engagement is assessed through two key components: BP, the measurable outcome of the task; and AE, the cognitive resources allocated to maintain focus (Pashler et al., 2001; Sarter et al., 2006). While interconnected, BP and AE are distinct constructs (Bruya & Tang, 2018); here, we provide physiological evidence supporting their differentiation.

To do so, we assessed BP via the HR and estimated AE from oculomotor activity (Ala et al., 2020; Gopher, 1973). These two metrics were used to explore the paradoxical decrease in selective tracking (rD) at high SNRs, a phenomenon driven by two counterintuitive effects: a reduction in target tracking (rT) even as the target becomes clearer, and an increase in masker tracking (rM) even as the masker becomes quieter. We found that BP, as measured by HR, has a limited ability to explain this relationship due to a ceiling effect at high SNRs. In contrast, AE, as measured by GV, effectively accounts for the decrease in selective neural tracking. The negative relationship we identified between oculomotor activity and task difficulty is consistent with prior studies and suggests that the reduction in rD is more accurately attributed to changes in AE rather than variations in BP (Contadini-Wright et al., 2023; Cui & Herrmann, 2023; Herrmann & Ryan, 2024). This finding aligns with previous work showing that selective attention modulates cortical responses without necessarily changing overall behavioral performance (Dai & Shinn-Cunningham, 2016).

Our findings also align with previous research indicating that increased oculomotor activity reflects reduced suppression of task-irrelevant psychological processes, impairing information processing such as selective neural speech perception (Abeles et al., 2020; Braga et al., 2016; Cui & Herrmann, 2023). More importantly, we provide a potential explanation for the reduced selective neural speech tracking in easier listening conditions that was also observed by (Das et al., 2018; Hauswald et al., 2022; Herrmann, 2024; Lesenfants et al., 2019). Notably, this negative impact of less suppressed oculomotor activity on selective neural speech tracking exists across SNRs, not only in the high SNR listening conditions (Fig. 3).

An interesting parallel is offered by (Gehmacher et al., 2024), who introduced the concept of ocular speech tracking, showing that oculomotor activity can temporally align with speech structure. While their paradigm differs from ours, the work highlights the potential of using oculomotor signals to study speech-related cognitive processes.

It is also worth mentioning studies that have demonstrated increased neural speech tracking in older populations and subjects with hearing impairment (Decruy et al., 2019; Decruy, Vanthornhout, et al., 2020). Our study offers an explanation for these observations: increased task difficulty in these subject populations elevates AE, thereby enhancing the selective neural speech tracking. To validate this hypothesis, we propose a follow-up study measuring differences in GV across populations or adjusting SNR to identify the threshold at which the selective neural tracking of target speech declines, comparing it with the threshold of normal-hearing subjects (approximately -1.6 dB in this study).

### 4.3 Modeling the interplay of speech intelligibility and attentional effort on neural speech tracking

In analyzing the interaction of various factors on predicting neural speech tracking, we found that both SI and GV have significant effects, while SNR shows no significant unique contribution after accounting for the other factors. Supplementary analyses and comparisons of TRFs of significant factors (SI and GV) revealed that SI influences both acoustic-related (TRF_50_, Ding & Simon, 2013) and attention-related components (TRF_100_, TRF_200_) (Ding & Simon, 2012b; Fiedler et al., 2019) of neural speech tracking, consistent with previous studies (Chen et al., 2023; Muncke et al., 2022). Notably, the modulation of early response (TRF_50_) may be attributed to the combined effect of SNR and SI, as shown in prior study, where a lower SNR at the same SI resulted in reduced TRF_50_ amplitude (Verschueren et al., 2020). In contrast, GV, as an indicator of AE, only modulates attention-related components, specifically the activation area of TRF_100_ and the intensity of TRF_200_. These components are closely associated with the top-down process of directing mental resources toward the target of interest (Fritz et al., 2007; Kong et al., 2014; Vanthornhout et al., 2019). These findings suggest that while SI affects multiple aspects of neural speech processing, GV’s influence is limited to the attentional mechanisms.

From the detailed analyses of the interaction among factors, we proposed a model based on our finding that AE and SI show a counterbalancing effect on neural speech tracking as SNR increases, with the dominant contribution shifting from SI to AE (Fig. 4). The proposed model is able to explain the widely observed non-linearity between task demands and neural speech tracking (Das et al., 2018, p. 201; Hauswald et al., 2022; Lesenfants et al., 2019), and also provides an explanation for the increased speech tracking in hard of hearing and aging populations (Decruy et al., 2019; Decruy, Vanthornhout, et al., 2020), for which the increased task difficulty could result in an increased AE.

There are several limitations to consider while interpreting our results. As EEG signals provide only a broad overview of cortical activity, complementary neuroimaging techniques would be needed to fully characterize the encoding of noisy speech in various cortical and subcortical auditory regions. Additionally, our measure of AE is indirect. While used extensively in the field (Ala et al., 2020; Gopher, 1973), GV is only an approximation of the cognitive resources that are used to maintain focus. Third, although we used two distinct types of background noise (babble and pedestrian), their effects on the SNR-SI psychometric curves were not significantly different in our data. These were included primarily to ensure that our findings generalize across different types of real-world masking and are not dependent on a specific noise profile. Future studies with larger, noise-type-balanced datasets may further explore whether specific masking conditions differentially interact with attentional engagement or intelligibility in shaping neural speech tracking. Fourthly, SI in Experiment 2 was not measured directly, but inferred from subject-specific SNR–SI functions obtained in a separate task (Experiment 1). While this cross-experimental mapping is common in studies of naturalistic speech (Etard & Reichenbach, 2019), we acknowledge that differences in stimulus structure and task demands could introduce variability. However, direct intelligibility assessments during continuous speech are difficult to implement without confounding effects from memory and attentional engagement, making our modeling approach a practical compromise. Finally, measuring BP via HR results in temporally sparse sampling of behavior, as it is possible that listeners lost focus between the repeated words. HR is meanwhile influenced by factors beyond attentional engagement, such as task difficulty and intelligibility. Despite its limitation in isolating the attention-directed performance from task demands, HR remains a practical and widely accepted behavioral measure in attention research. Future study is needed to explore more direct methods to measure cognitive load and behavioral performance, and to expand these findings to the aging and hard-of-hearing populations.

### 4.4 Comparison between attentional effort and listening effort

In this work, we used two metrics of attentional engagement (AE and BP) to investigate the role of attention in modulating neural speech tracking and to assess their ability to explain the reduction in rD under favorable listening conditions. As previously defined, AE involves the cognitive resources and motivation required to focus on target speech while ignoring distractors (Pashler et al., 2001; Sarter et al., 2006). Our findings indicate that AE positively predicts rD but decreases as target speech becomes easier to follow, ultimately explaining the observed reduction in rD with increasing SNR.

A related but distinct concept is listening effort (LE), which refers to the cognitive resources involved in performing listening tasks (Pichora-Fuller et al., 2016). While both AE and LE pertain to mental resource allocation, they differ fundamentally and may overlap in certain contexts. First, LE is not necessarily tied to a specific attentional focus in the presence of distractors. Second, AE is not exclusive to listening tasks (Sarter et al., 2006; Westbrook & Braver, 2015). In the modeling of attentional engagement in effortful auditory processing (Strauss & Francis, 2017), AE is required for object selection, where attention is engaged in selecting a target sound from competing inputs (Sarter et al., 2006; Shinn-Cunningham, 2008; Shinn-Cunningham & Best, 2008). In contrast, LE becomes particularly relevant when additional coping mechanisms, such as filling in missing speech, are needed to reconstruct degraded input (McGarrigle et al., 2017; Pichora-Fuller et al., 2016; Shinn-Cunningham, 2008; Shinn-Cunningham & Best, 2008).

In attentive listening tasks with distractors, where the interplay of attentional effort and listening effort becomes complex and overlapped (Strauss & Francis, 2017), LE is widely measured by pupil response (Ala et al., 2020; Kuchinsky et al., 2014; Winn et al., 2018; Zekveld et al., 2018), despite concerns about its efficacy in capturing the multidimensional nature of LE (Alhanbali, 2019). Some studies suggest that pupil response during such tasks may reflect attention modulation (Hoeks & Levelt, 1993; Koelewijn et al., 2015). However, AE is more commonly measured through oculomotor activity due to its anatomical overlap with attention-related cortical areas (Corbetta et al., 1998) and supporting physiological evidence (Abeles et al., 2020; Braga et al., 2016; Contadini-Wright et al., 2023; Cui & Herrmann, 2023; Gopher, 1973; Kobald et al., 2019; Šabić et al., 2020). Moreover, compared to pupil response, oculomotor factors are considered a more reliable index of instantaneous auditory attention, cognitive load, and cognitive control strategy (Contadini-Wright et al., 2023; Cui & Herrmann, 2023; Herrmann & Ryan, 2024). In our study, LE, as measured by pupil dilation, shows reduced variability in the high-SNR range (Supplementary Fig. S1A) and does not account for the observed reduction in rD. Similarly, Müller et al. (2019) demonstrated that when pupillary-based LE was modulated by altering speech rate, without directly affecting AE due to the absence of competing stimuli or distractors, neural speech tracking remained unchanged. This study further emphasizes the distinction between AE and LE and cautions against using pupillometry as a measure of attentional engagement, as it is influenced by multiple factors involved in listening tasks beyond AE.

It remains unclear whether the classical inverted U-shaped relationship between LE and task demands (SNR) can be extended to AE, as empirical evidence is currently limited. Importantly, Herrmann and Ryan (2024) observed that oculomotor activity continued to decrease under “impossible” listening conditions, while pupil-dilation-based LE showed a non-monotonic pattern consistent with the “giving up” phenomenon when task demands exceeded cognitive capacity. In our study, when speech became nearly unintelligible at around -12 dB SNR, we observed a slight decrease in AE, accompanied by reduced pupil dilation (Fig. 3B and Supplementary Fig. S1A), suggesting a possibly similar U-shape trend. Further research involving more extreme listening conditions is needed to rigorously examine the relationship between AE and task demands.

## Supplementary Material

Supplementary Material

## Data Availability

The dataset, which includes anonymized segmented EEG recordings, trial-level objective and perceptual-related factors, neural metrics (e.g., SNR, SI, rT, rM), and standardized speech envelopes, together with the analysis code used to generate all figures and perform statistical modeling, is available at the following GitHub repository: https://github.com/naplab/AAD_INT
