# Supplementary material for: Neural speech tracking in noise reflects the opposing influence of SNR on intelligibility and attentional effort
Source: Imaging Neurosci (Camb). 2025 Aug 28;3:IMAG.a.126. doi: 10.1162/IMAG.a.126 (PMC12395281; doi:10.1162/IMAG.a.126)
Supplement: Supplementary Material [file IMAG.a.126_supp.pdf]

Supplementary Material

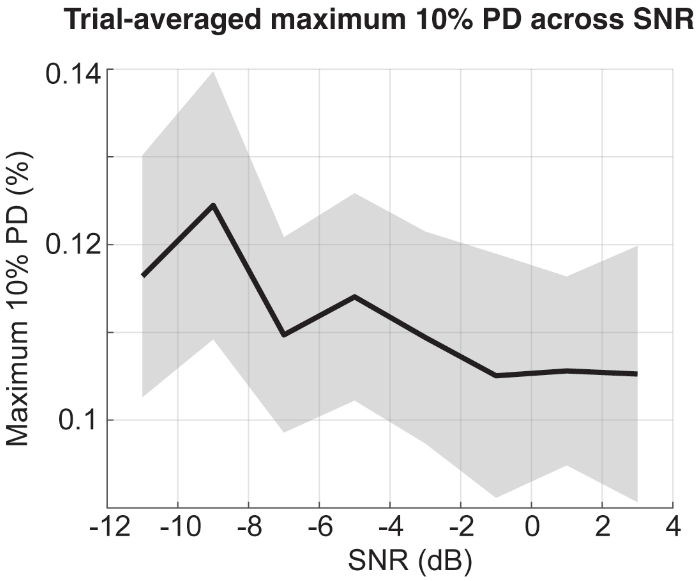

Supplementary Fig. 1. Pupil dilation (PD) vs. SNR. Trial-averaged peak PD (top 10% within-trial values) plotted against SNR, averaged across subjects. Shaded areas indicate ±1 SEM. PD was baseline-corrected relative to the 1-second period preceding trial onset and expressed as a relative change ratio.
